# POTENTIAL MENTAL BENEFITS OF REMOTE EXPRESSIVE ARTS PROGRAM IN OLDER ADULTS WITH MILD COGNITIVE IMPAIRMENT

**DOI:** 10.1093/geroni/igae098.3322

**Published:** 2024-12-31

**Authors:** Yuting Luo, Hong Li

**Affiliations:** Fujian Medical University, Fuzhou, Fujian, China (People’s Republic); Fujian Medical University, Fuzhou, Fujian, China (People’s Republic)

## Abstract

The use of expressive arts program (EAP) in mild cognitive impairment may positively impact the psychological well-being. A future direction for EAP may be to explore a new channel for MCI patients to participate via the mobile internet, as this could improve accessibility. The study explores the impact of a remote expressive arts program (rEAP) on the mental health of older adults with mild cognitive impairment (MCI). Conducted through a two-arm, single-blinded, pilot, randomized controlled trial, participants were assigned to either a 12-week rEAP or health education (HE) group. Pre- and post-treatment psychological evaluations were conducted. Results from 73 MCI patients showed significant improvements in anxiety mood (Self-Rating Anxiety Scale, P=0.032) and potential benefits in subjective well-being, depression, self-esteem, and self-efficacy in the rEAP group compared to the HE group. Qualitative interviews with rEAP participants highlighted enthusiasm, feasibility, usability, and content satisfaction. Positive cognitive and mental benefits were reported, with themes including enhanced cognitive function perception, relaxation, creativity, self-confidence, and social engagement. The findings suggest that a 12-week rEAP can enhance the psychological well-being of older adults with MCI, indicating its feasibility and applicability for this population. The study underscores the significance of rEAP as a feasible intervention leveraging mobile internet to improve mental health in older adults with MCI.

